# Supplementary material for: Engineered Graphene Quantum Dots as a Magnetic Resonance Signal Amplifier for Biomedical Imaging
Source: Molecules. 2023 Mar 3;28(5):2363. doi: 10.3390/molecules28052363 (PMC10005761; doi:10.3390/molecules28052363)
Supplement: Supplementary file 1 [file molecules-28-02363-s001.zip › molecules-2235563-supplementary.pdf]

Supplementary Material

Engineered Graphene Quantum Dots as a Magnetic Resonance Signal Amplifier for Biomedical Imaging

Zhongtao Li [1], Guiqiang Qi [1], Guangyue Shi [1], Meng Zhang [2], Haifeng Hu [3] and Liguo Hao [1,*]

[1] Department of Molecular Imaging, School of Medical Technology, Qiqihar Medical University, Qiqihar 161006, China
[2] Animal Laboratory Center, Qiqihar Medical University, Qiqihar 161006, China
[3] Department of MRI, The Second Affiliated Hospital of Qiqihar Medical University, Qiqihar 161006, China
* Correspondence: haoliguo@qmu.edu.cn

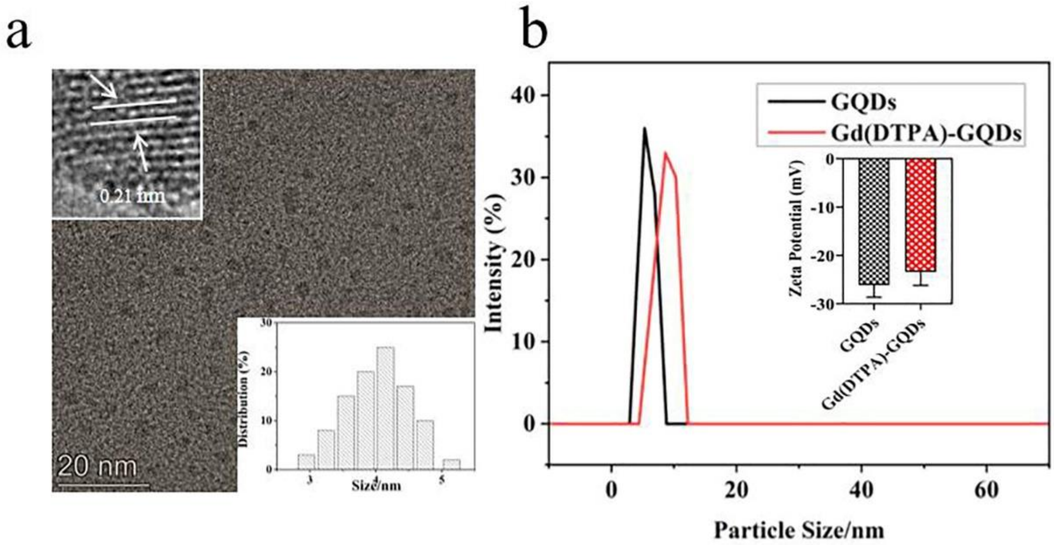

Figure S1. Structural characterization: (a) TEM image and size distribution of Gd(DTPA)-GQDs sample (Inset is the HR-TEM of Gd(DTPA)-GQDs). (b) Hydrodynamic diameters and zeta potentials of GQDs and Gd(DTPA)-GQDs.

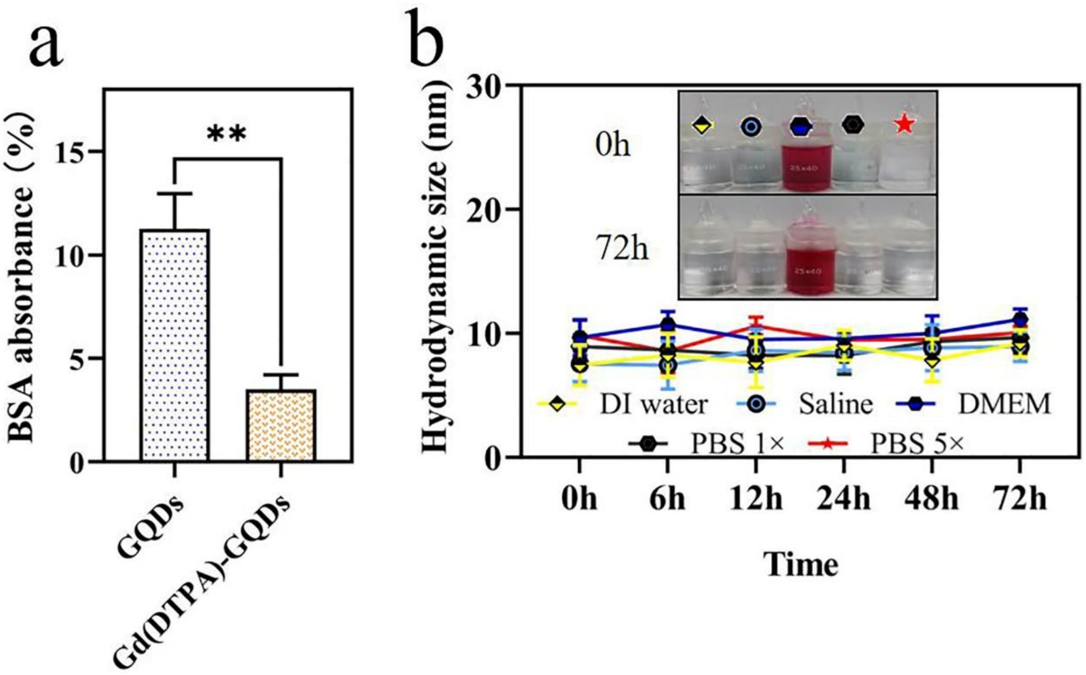

Figure S2. (a) BSA adsorbance amounts of GQDs and Gd(DTPA)-GQDs ($n = 3$, $^{**}p < 0.01$). (b) Stability of Gd(DTPA)-GQDs nanocomposites in different biological solutions (Inset is digital photographs of Gd(DTPA)-GQDs in several solutions such as DI water, saline, DMEM, PBS 1×, and PBS 5×).

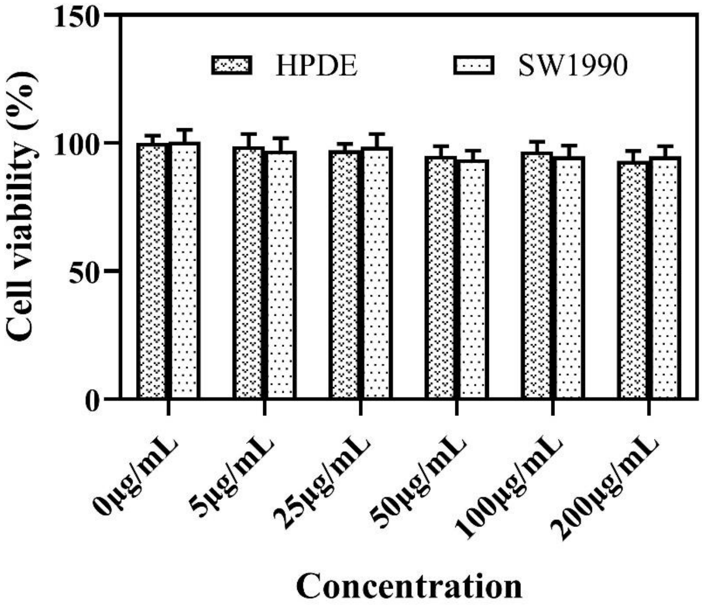

Figure S3. Cytotoxicity against HPDE cells and SW1990 cells after incubation with different concentrations of various GQDs for 24 h.

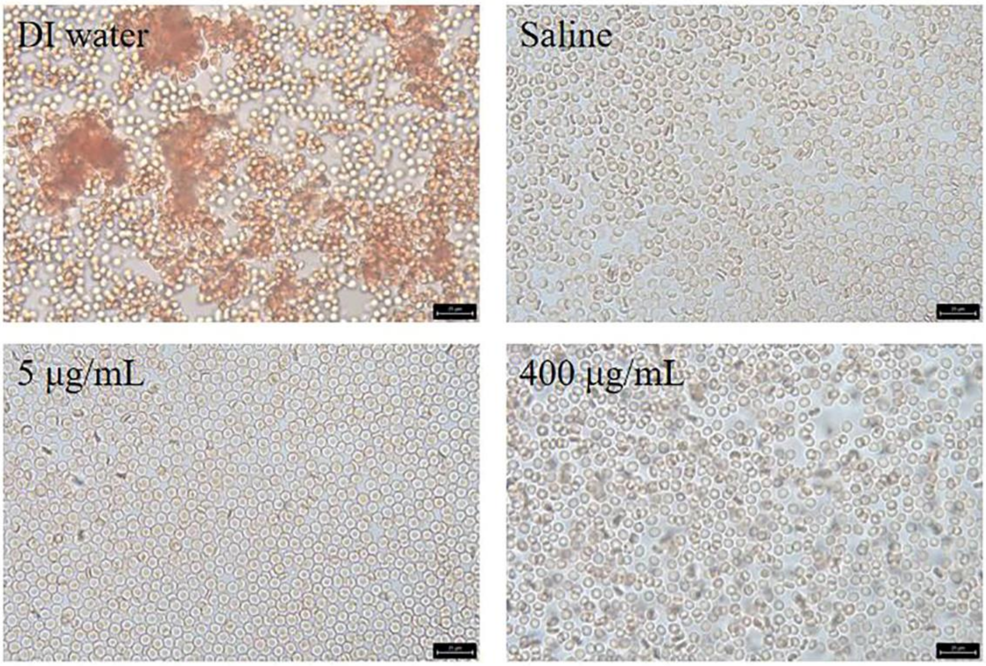

Figure S4. Morphology of human red blood cells upon treatment with saline, DI water and Gd(DTPA)-GQDs (5, 400 μg/mL) for 4 h.

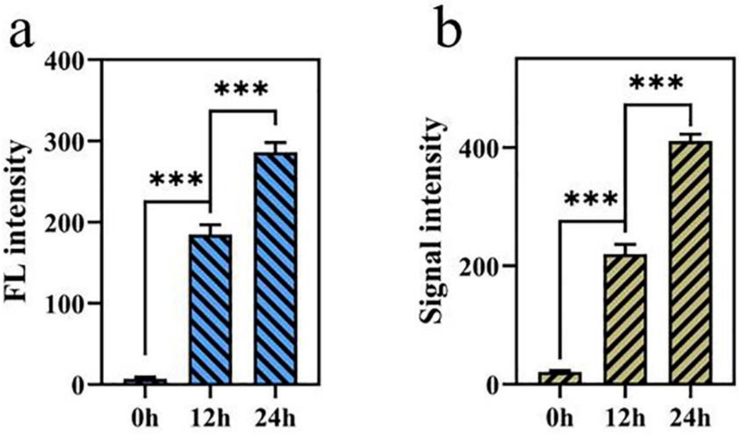

Figure S5. Mean fluorescence intensity (a) and MRI signal intensity (b) of SW1990 cells after incubation with Gd(DTPA)-GQDs at different times. ($***p < 0.001$).
